# Simplifying causal gene identification in GWAS loci

**DOI:** 10.1101/2024.07.26.24311057

**Published:** 2024-07-29

**Authors:** Marijn Schipper, Jacob Ulirsch, Danielle Posthuma, Stephan Ripke, Karl Heilbron

**Affiliations:** 1 Department of Complex Trait Genetics, Center for Neurogenomics and Cognitive Research, Amsterdam Neuroscience, Vrije Universiteit Amsterdam, Amsterdam, The Netherlands; 2 Program in Medical and Population Genetics, Broad Institute of Harvard and MIT, Cambridge, MA, USA.; 3 Stanley Center for Psychiatric Research, Broad Institute of MIT and Harvard, Cambridge, Massachusetts, USA; 4 Illumina Artificial Intelligence Laboratory, Illumina, San Diego, CA, USA.; 5 Department of Child and Adolescent Psychiatry and Pediatric Psychology, Section Complex Trait Genetics, Amsterdam Neuroscience, Vrije Universiteit Medical Center, Amsterdam, The Netherlands; 6 Department of Psychiatry and Psychotherapy, Charité – Universitätsmedizin Berlin, Berlin, Germany; 7 German Center for Mental Health (DZPG), partner site Berlin/Potsdam, Berlin, Germany

## Abstract

Genome-wide association studies (GWAS) help to identify disease-linked genetic variants, but pinpointing the most likely causal genes in GWAS loci remains challenging. Existing GWAS gene prioritization tools are powerful, but often use complex black box models trained on datasets containing unaddressed biases. Here we present CALDERA, a gene prioritization tool that achieves similar or better performance than state-of-the-art methods, but uses just 12 features and a simple logistic regression model with L1 regularization. We use a data-driven approach to construct a truth set of causal genes in 406 GWAS loci and correct for potential confounders. We demonstrate that CALDERA is well-calibrated in external datasets and prioritizes genes with expected properties, such as being mutation-intolerant (OR = 1.751 for pLI > 90%, P = 8.45×10^−3^). CALDERA facilitates the prioritization of potentially causal genes in GWAS loci and may help identify novel genetics-driven drug targets.

Retrospective analyses have found that drugs are more likely to be approved by regulators if there is human genetic evidence supporting a connection between the drug target and indication^1,2^. Indeed, 63% of drugs approved by the FDA between 2013 and 2022 were supported by human genetic evidence^3^, and the relative success of genetically-supported drug targets has not decreased over time^4^.

Genome-wide association studies (GWAS) are a valuable tool for identifying associations between diseases and genetic variants. However, the vast majority of GWAS loci contain multiple genes and the vast majority of GWAS variants do not alter protein coding sequences. A key challenge in using GWAS data to identify potential drug targets is determining which genes are affected by disease-associated variants. Several gene prioritization tools have been developed to identify the most likely effector gene for a given GWAS signal such as Ei^5^, FLAMES^6^, and L2G^7^. These three tools all model the probability that each gene in a GWAS locus is a causal gene using 1) XGBoost, 2) a truth set of causal and non-causal trait-gene pairs, and 3) a variety of features. The FLAMES study performed a head-to-head comparison of these methods and found that FLAMES outperformed L2G and Ei, which in turn outperformed cS2G^6^.

There are two main drawbacks to current gene prioritization tools. First, XGBoost models are challenging to interpret. While regression methods estimate a single effect size for each feature, the contribution of a given feature in an XGBoost model depends on the value of other variables. EI, FLAMES, and L2G all use more than 45 features—many of which are highly collinear—further complicating model interpretation. Second, models need to be trained on a ground truth dataset. Expert-curated causal genes have been shown to be biased towards genes in close proximity to GWAS hits and biased towards genes affected by coding credible set variants^7^. Although some methods try to mediate this by using a data-driven strategy for constructing ground-truth datasets^6^, none actively correct for potential sources of bias.

To address these issues we present a novel gene prioritization tool, CALDERA (CALling Disease-RelAted genes). CALDERA uses a logistic regression model with an L1 penalty (LASSO), a small number of features, a data-driven truth set, and covariates to account for biases in this truth set. We show that CALDERA achieves state-of-the-art performance whilst using a simpler and more interpretable model.

## Results

### Defining causal genes

We constructed a set of putatively causal (and non-causal) trait-gene pairs using SuSiE^8^ credible sets for 19 independent (genetic correlation < 0.2) UK Biobank traits^9^. Within a given trait, we defined causal genes as those that were 1) affected by a fine-mapped non-synonymous variant (posterior inclusion probability [PIP] > 50%) and 2) within 300kb of a separate non-coding credible set (no non-synonymous variant PIP > 50%). We defined non-causal genes as all other genes within 300kb of these non-coding credible sets. This resulted in a set of 406 putatively causal genes and 4,358 putatively non-causal genes across 19 independent traits.

### Model performance using the full feature set

**Figure 1.**
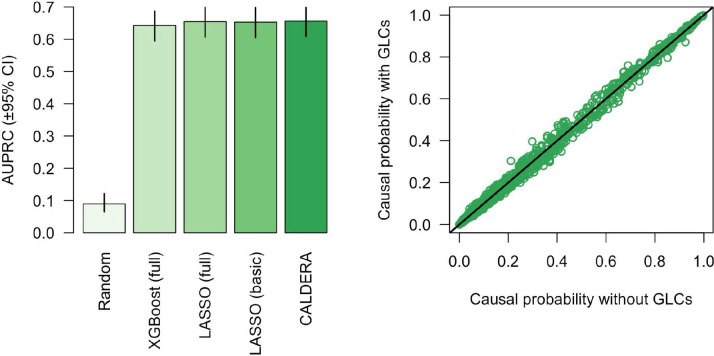
**A.** Area under the precision-recall curve (±95% confidence intervals) for models predicting causal and non-causal genes for 19 independent traits. Full = the full set of 52 gene prioritization features, basic = the basic set of 12 gene prioritization features. **B.** Causal probability estimated by LASSO models using the basic feature set with (y-axis) and without (x-axis) correcting for gene-level covariates (GLCs). Each point represents a single trait-gene pair. The solid black line represents an equivalent value for the x- and y-axis variables.

Next, we trained LASSO and XGBoost models to predict causal gene status using a set of 52 features derived from: distance to GWAS lead variant, non-synonymous variant PIP (all <50% by definition), number of local genes, activity-by-contact (ABC)^10^, enhancer-promoter correlation^11–13^, eQTL colocalization^14^, MAGMA^15^, promoter capture Hi-C (PCHi-C)^16,17^, summary data-based Mendelian randomization (SMR)^18^, transcriptome-wide association studies (TWAS)^19^, DEPICT^20^, NetWAS^21^, and polygenic priority score (PoPS)^9^. To assess model performance, we trained the models in a nested leave-one-trait-out cross-validation framework. Model performance in held-out traits was similar for both LASSO (Figure 1A, Figure S1, area under the precision-recall curve [AUPRC] = 65.3%, 95% confidence interval [CI] = 60.6% to 69.8%) and XGBoost (AUPRC = 64.4%, 95% CI = 59.6% to 68.9%). This suggests an absence of strong feature-feature interactions and non-linear relationships between causal gene status and features (after feature transformation, see Methods). Due to similar model performance, we proceeded using the simpler LASSO model.

### Model performance using a basic set of features

Applying these models to obtain predictions for a new GWAS of interest requires running a wide range of pipelines to construct the full feature set. We therefore tested the performance of a LASSO model that only used a basic set of features: distance to GWAS lead variant, non-synonymous variant PIP, number of local genes, MAGMA, and PoPS. Despite the large reduction in the number of features, performance in held-out traits was similar for both the full feature set (AUPRC = 65.3%, 95% CI = 60.6% to 69.8%) and the basic feature set (AUPRC = 65.2%, 95% CI = 60.4% to 69.6%, Figure 1A). We therefore proceeded using the basic feature set.

### Accounting for bias

Genes nearest to GWAS lead variants (a proxy for causal genes) are more likely to be mutation-intolerant than genes nearest to matched control variants^22^. However, we defined causal genes using fine-mapped coding variants and, therefore, our set of causal genes was enriched for being mutation-tolerant (Fisher’s exact test for pLI < 10%: OR = 1.725, 95% CI = 1.348 to 2.227, P = 6.0×10^−6^). A key strength of our models is the ability to account for sources of bias such as this. As such, we included a set of gene-level covariates pertaining to mutational constraint, gene length, and enhancer length. When generating predictions in the test set, covariate effects were removed by setting covariate values to their mean. Including covariates did not substantially affect model performance (Figure 1B, AUPRC = 65.3%, 95% CI = 60.5% to 69.7%). After covariate correction, however, predicted causal probabilities > 20% decreased by an average of 3.0% (Figure 1B). This suggests that these predictions were inflated due to biases in the training data. We therefore performed all downstream analyses with the models trained using gene-level covariate bias correction.

### Model interpretation

**Figure 2.**
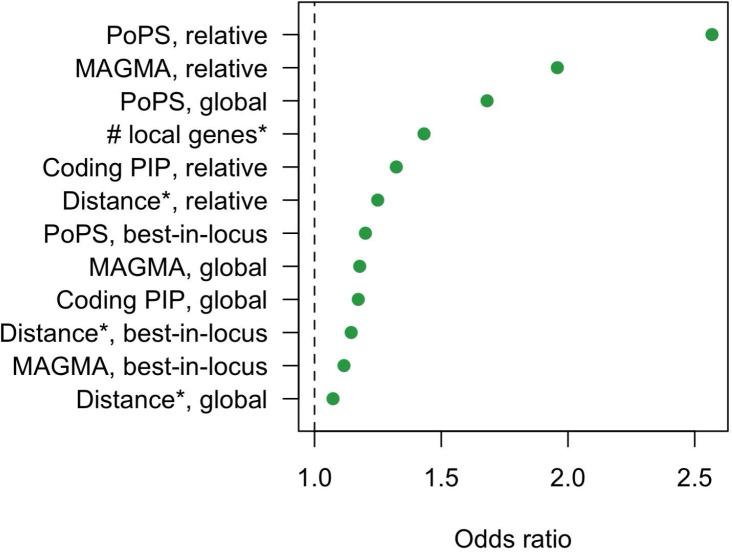
Standardized coefficients (feature standard deviation = 1) from a LASSO model predicting causal gene status for 19 independent traits using the basic feature set and gene-level covariates. PoPS = Polygenic Priority Score, MAGMA = MAGMA z-score, Distance = distance between gene and GWAS lead variant, Coding PIP = non-synonymous variant posterior inclusion probability. Best-in-locus = a binary feature denoting the gene with the largest global value in a locus (excluding ties), relative = the global value for a gene subtracted by the best global value in the locus. Asterisks (*) denote features that have been transformed, see Online Methods for details. For distance and number of local genes, these transformations included multiplying values by −1 to ensure that increasing feature values leads to increased predicted causal probability.

**Figure 3.**
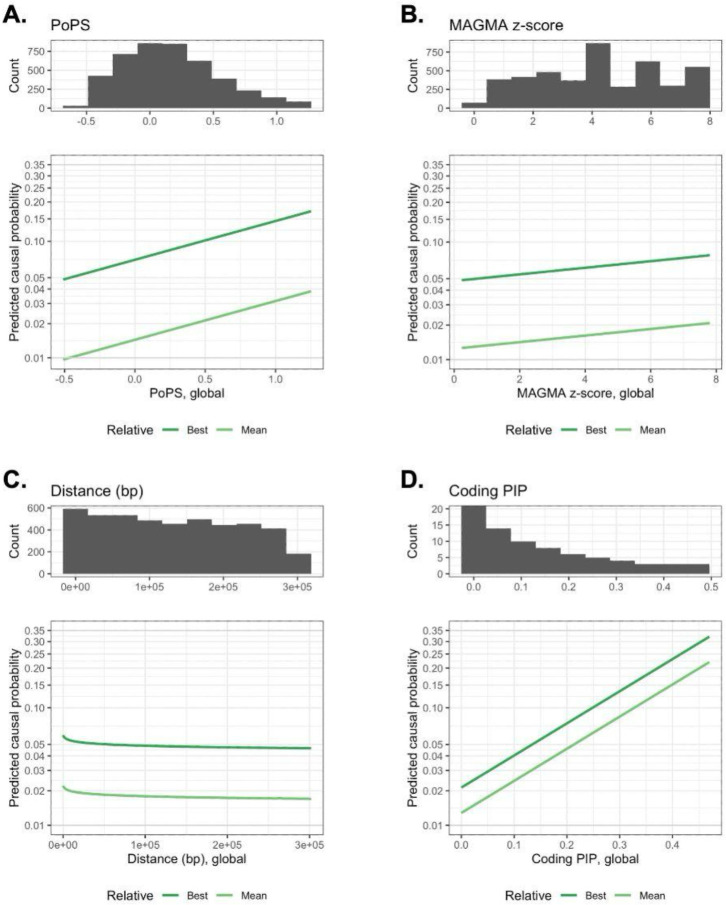
Relationship between predicted causal gene probability and (**A**) PoPS, (**B**) MAGMA z-score, (**C**) distance between gene and GWAS lead variant (in base pairs), and (**D**) non-synonymous credible set variant posterior inclusion probability (PIP). The lower y-axis represents the probability that a given gene is causal for a given trait and has been logit-transformed. The x-axis represents global feature values ranging from the 5th to the 95th percentile (except for coding PIP, which ranges from the 0th to the 100th percentile). Histograms showing global feature distribution are plotted at the top of each panel. For coding PIP, the histogram y-axis was truncated at 20 for clarity (count of first bin = 4,787). Dark green lines represent genes with the best focal feature value in the locus. Light green lines represent genes with the average focal feature value in the locus. All other features were set to their mean, leading to low overall probabilities. Although transformed distances were used to train the model, untransformed values are presented to facilitate interpretation. We imputed missing MAGMA z-scores to the median (4.223), resulting in a spike in the distribution.

We trained a LASSO model on all 19 independent traits using the basic feature set and gene-level covariates. To compare the contribution of each feature to the model, we plotted their coefficients (Figure 2), standardized to represent an increase of one standard deviation (SD). For all gene prioritization methods, relative features had larger standardized effects than global features, suggesting that relative value within a locus is more informative than absolute value. For relative and global features, there was a consistent rank ordering of gene prioritization methods (PoPS > MAGMA > coding PIP > distance). To help further visualize predicted feature effects, we plotted model-predicted causal probability across a wide range of actual feature values (Figure 3).

### Calibration

**Figure 4.**
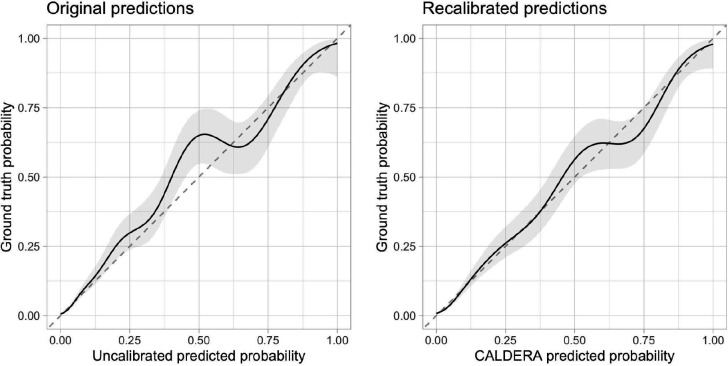
Calibration plots before (**A**) and after (**B**) recalibration. The x-axis represents model predicted probability in held-out trait data and the y-axis represents the ground truth causal probability. The solid lines represent the fitted value from generalized additive models with shaded areas representing 95% confidence intervals. The dashed lines represent perfect calibration.

Model predictions for held-out traits were largely well-calibrated, although predictions between approximately 35% and 55% were slightly conservative (Figure 4A). Local recalibration (Figure 4B, see Online Methods) did not negatively affect model performance (Figure 1A, AUPRC = 65.5%, 95% CI = 60.7% to 69.9%) and more accurately reflected the probability that a given gene is causal for a given trait. Putting all previous results together, we present CALDERA: a LASSO model trained on a data-driven set of causal and non-causal genes using a basic set of 12 features—as well as a set of gene-level covariates to correct for bias—followed by local recalibration.

### CALDERA recovers known characteristics of GWAS genes

Previous work has shown that putative GWAS genes are more likely to be mutation-intolerant (pLI > 90%), more likely to be transcription factors, and have a larger number of unique transcription start sites (TSSs)^22^. Even though CALDERA was trained on a set of causal genes that was biased towards being mutation-tolerant, putatively causal CALDERA genes (predicted causal probability > 50% for any trait, n = 149) were more likely to be mutation-intolerant than the remaining 2,043 genes in significant GWAS loci (22.8% versus 14.7%, P = 8.45×10^−3^). We found similar results for the proportion of transcription factors (10.7% versus 6.4%, P = 0.044) and the average number of unique TSSs (6.9 versus 2.7, P = 1.35×10^−10^). These results demonstrate that CALDERA can effectively overcome biases in its training dataset. It prioritizes genes with expected properties and successfully recovers causal GWAS genes, even when the training set is under enriched in genes with known causal GWAS gene characteristics.

### No evidence of bias due to causal genes shared between traits

Although we only used traits with a global genetic correlation coefficient < 20%, 31 genes were causal for multiple independent traits. We repeated our analyses using a set of 189 non-shared causal genes (2,053 non-causal genes). We observed little difference in AUPRC when using models trained in the dataset without shared causal genes (Figure S3, AUPRC = 64.5%, 95% CI = 57.5% to 71.0%). Furthermore, there was negligible difference between a logistic regression model (AUPRC = 65.2%, 95% CI = 60.4% to 69.7%) and a generalized linear mixed model using the causal gene as a random effect (AUPRC = 65.2%, 95% CI = 60.4% to 69.7%). These results suggest that CALDERA performance was not substantially inflated due to shared causal genes shared across traits.

### Benchmarking performance against L2G

**Figure 5.**
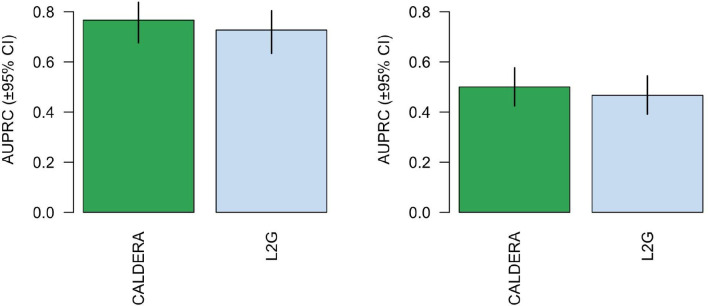
Area under the precision-recall curve (±95% confidence intervals) for CALDERA and L2G model predictions in **A)** the Open Targets ground truth dataset or **B)** a ground truth dataset derived from burden tests of rare coding variants in the UK Biobank.

L2G is a popular gene prioritization tool that has been shown to outperform other published methods^6^. We therefore compared the performance of CALDERA and L2G in two external gold standard datasets of causal and non-causal trait-gene pairs. First, we used the Open Targets gold standard dataset. Even though L2G was trained on this dataset, AUPRC was higher for CALDERA (Figure 5A, AUPRC = 76.6%, 95% CI = 67.6% to 83.7%) than for L2G (AUPRC = 72.7%, 95% CI = 63.4% to 80.4%). Second, we used a gold standard dataset derived from burden tests of rare coding variants in the UK Biobank. Again, AUPRC was non-significantly higher for CALDERA (Figure 5B, AUPRC = 50.0%, 95% CI = 42.4% to 57.7%) than for L2G (AUPRC = 46.7%, 95% CI = 39.2% to 54.4%). CALDERA predictions were well-calibrated in both gold standard datasets (Figure S2). These results demonstrate that CALDERA achieves state-of-the-art performance while using a simpler and more interpretable model.

## Discussion

In this work we have developed CALDERA, a simple tool for prioritizing genes in GWAS loci. CALDERA is interpretable, accounts for bias, and achieves state-of-the-art prediction performance.

Since CALDERA uses a LASSO model, it is easier to interpret than XGBoost-based models. An increase in a given feature leads to a linear increase in the log odds that a given gene is causal. As shown in Figure 3, this makes it simple to visualize and understand the relationship between features and CALDERA’s predicted causal probabilities. By contrast, this is not possible for XGBoost models, where the effect of increasing a given feature is typically dependent on the values of other features. CALDERA’s interpretability is further facilitated by the fact that it only uses 12 features to generate predictions—far fewer than FLAMES (47 features), L2G (51 features), and EI (154 features). We have provided code to generate the 12 CALDERA features—as well as CALDERA predicted causal gene probabilities—using only a PoPS output file, a MAGMA output file, and a file containing credible set information (see Code Availability). Overall, the predictions made by CALDERA are significantly easier to understand than those of other current methods.

To account for biases in the CALDERA truth set, we used two strategies. First, we used a data-driven truth set, rather one that was manually curated by human experts. The L2G study found that some distance and coding features performed much better in manually-curated datasets than in data-driven datasets derived from CHEMBL^7^. This suggests that many of these causal genes were selected precisely because of their close proximity to a GWAS signal or due to a credible set coding variant. Second, we carefully considered potential sources of bias in our truth set based on how our causal genes were selected and attempted to account for these biases using gene-level covariates. To our knowledge, CALDERA is the first gene prioritization tool that attempts to actively correct for truth set biases. Failing to account for these biases led to systematic inflation of predicted causal probabilities greater than ~20% (Figure S1). Gene prioritization tools that do not correct for biases may suffer from similarly inflated predictions. Even though the CALDERA truth set was enriched for mutation-tolerant genes, CALDERA-prioritized genes were enriched for mutation intolerance, as expected^22^.

Despite using a simpler model, CALDERA AUPRCs were higher than the L2G AUPRCs—even in the L2G training dataset (Open Targets, Figure 5). The CALDERA model placed a large emphasis on PoPS and MAGMA (Figure 2), which are not present in the L2G model. Unlike most commonly used gene prioritization features, PoPS is a similarity-based method that integrates genome-wide information^9^. The orthogonality of PoPS-derived information likely explains much of its large contribution to the CALDERA model. MAGMA z-scores capture the amount of statistical signal near a given gene body and might partially function as an alternative distance metric (r = 36% for global MAGMA and distance). Overall, these results suggest that the simplified CALDERA feature set sufficiently replaced the larger L2G feature set whilst maintaining state-of-the-art performance.

There are some limitations to this study. We assumed that genes bearing a coding variant with PIP > 50% are causal for a given trait. While a variant with PIP = 50% should only have a 50% probability of being the causal variant, this probability should be much higher for coding variants^23^ and 73% of our causal genes had a coding variant PIP > 90%.

More importantly, we also assumed that all non-coding credible sets within 300kb of one of these genes also acts through the same causal gene. Reprocessing published data^22^, we found that 87% of cis-eQTLs lie within 100kb of their effector gene and that the percentage of effector genes drops steeply as distance increases further (Figure S4). We found similar results for the distance between GWAS hits and their nearest gene, a proxy for the causal gene (Figure S4). By definition, the distance between GWAS hits and their true effector genes must be larger. Nevertheless, these data and others^24^ suggest that, beyond a certain distance, the probability of being a causal gene begins to decrease in an exponential-like fashion. As such, distal causal genes in the CALDERA truth set may be less reliable than more proximal genes.

At the same time, there are well-documented examples where the causal gene lies further than 300kb from the credible set^25^. Nevertheless, CALDERA showed good calibration (Figure S2) in both the Open Targets and ExWAS gold standard datasets, which used 500kb and 750kb windows, respectively. This suggests that CALDERA can be robustly applied to larger locus definitions than the ones on which it was trained.

Another limitation of CALDERA is that it was trained on features computed using in-sample linkage disequilibrium (LD) from one cohort (UK Biobank). Using out-of-sample LD reference panels can lead to errors in all three sources of CALDERA features—PoPS, MAGMA, and fine-mapped credible sets. Additionally, GWASes that meta-analyze multiple cohorts commonly have heterogeneous sample sizes across variants. This leads to misspecified credible set PIPs^26^, although MAGMA and PoPS can process variant-specific sample sizes and are therefore more robust. Prior to using CALDERA, we therefore advise the use of tools to check for discrepancies between GWAS summary statistics and the LD reference panel and the removal of failing variants or loci^26,27^.

Finally, because LD patterns differ across ancestral populations, CALDERA predictions may not be well-calibrated in non-European populations. Unfortunately, this is challenging to test at present. Identifying the 406 causal trait-gene pairs in the CALDERA truth set required GWAS data for 19 independent traits, each of which was performed on hundreds of thousands of individuals. Fortunately, this is likely to be possible in the near future thanks to biobank-scale initiatives in individuals of diverse ancestries, such as All of Us^28^.

In conclusion, we present CALDERA, a model that allows for accurate and interpretable GWAS gene prioritization. CALDERA performance is similar to other state-of-the-art methods, but uses a more-interpretable model, requires fewer input features, and corrects for potential biases. Leveraging CALDERA could aid the prioritization of novel causal disease genes and the identification of novel drug targets.

## Online Methods

### Variant-to-gene evidence

We extracted predictive features for all trait-gene pairs from the original PoPS study^9^. These included distance to GWAS lead variant, non-synonymous variant PIP, ABC^10^, enhancer-promoter correlation^11–13^, eQTL colocalization^14^, MAGMA^15^, PCHi-C^16,17^, SMR^18^, TWAS^19^, DEPICT^20^, NetWAS^21^, and PoPS^9^. We only included canonical ENSGIDs. To determine the number of local genes we included all GENCODE v44^29^ genes within 300kb of the focal credible set.

### Creating a set of causal and non-causal trait gene pairs

To define a set of causal (and non-causal) trait-gene pairs, we used SuSiE credible sets for 39 independent UK Biobank GWASes^30^ (Table S1 for independent traits). To minimize the risk of errors in SuSiE fine-mapping, we subsetted to the top 5 credible sets within each region. We identified credible sets containing a non-synonymous variant with PIP > 50% (“coding credible sets”) and the affected gene (“coding genes”). We designated the remaining credible sets as “non-coding credible sets” (no non-synonymous variant with PIP > 50%). We subsetted to non-coding credible sets within 300kb of a single coding gene for the same trait and with a maximum credible set width of 400kb. We extracted all protein-coding genes within 300kb of each of these non-coding credible sets, assigned the nearby coding gene as a “causal gene”, and assigned all others as “non-causal genes”. As such, the maximum locus size was 1Mb—a 400kb credible set plus 300kb on either side. We chose a window of 300kb because previous work has shown that 90% of eQTLs are found within 130kb of their causal gene and 90% of GWAS hits are found within 108kb of the nearest gene (a proxy for the causal gene)^22^. We removed loci containing fewer than two genes and removed traits with fewer than five causal genes (19 traits remained). Finally, we joined variant-to-gene mapping evidence to this causal gene dataset by trait and gene.

### Feature engineering and missing data imputation

We left data untransformed for PoPS^9^, MAGMA z-scores^15^, coding PIPs, Andersson and Ulirsch enhancer-promoter correlations^11,13^, Jung and Javierre PCHi-C interaction scores^16,17^, DEPICT z-scores^20^, NetWAS scores^21^, and NetWAS Bon scores^21^. For TWAS^19^, we used the absolute value of the z-score. We log_10_-transformed Roadmap enhancer-promoter correlations^12^, eQTL colocalization posterior probabilities^14^, ABC-Max scores^10^, and SMR^18^ P values. For distance-related variables (GWAS lead variant to gene body, GWAS lead variant to transcription start site [TSS]), we added 1 kilobase prior to log_10_-transformation. We used a logit_10_ transformation to convert the inverse of the number of local genes (*i.e.* the prior probability that a gene is causal) to a log_10_ odds scale. For all log_10_- and logit_10_-transformed variables, we imputed missing or zero values to the minimum non-missing and non-zero value (except missing SMR P values, which were imputed to 1). For all other variables, missing data were imputed to 0 (except MAGMA z-scores, which were imputed to their median value). We multiplied the transformed SMR and distance-related values by −1 to ensure a positive relationship with causal gene status.

### Relative and best-in-locus features

Within each locus, we assigned the gene with the largest value for a given feature as the “best-in-locus”, excluding ties. In addition, we constructed “relative scores” within each locus by subtracting a gene’s value by the largest local value. This resulted in a full set of 52 features: 17 groups multiplied by 3 types (global, best-in-locus, and relative), as well as the number of local genes.

### Basic feature set

We aimed to create a minimal set of features that would yield a similar AUPRC to the full feature set. We selected distance, coding PIP, and the number of local genes based on their importance in the L2G and FLAMES models. We selected PoPS since, unlike the aforementioned features, it integrates information from outside of the focal locus. Because PoPS requires MAGMA results as an input, we also included MAGMA.

### Gene-level covariates

To account for bias introduced by our process for selecting causal genes, we curated a set of gene-level covariates^22^ related to genetic constraint (probability of being loss-of-function intolerant [pLI]^31^ and heterozygote selection coefficient [hs]^32^), gene length (total and coding sequence), and enhancer length (from ABC^33^ and Roadmap^12^ datasets). We log_10_-transformed all covariate values and imputed missing values to the minimum non-missing value except pLI (missing values imputed to 0.5) and hs (missing values imputed to the maximum non-missing value). We multiplied transformed pLI and hs values by −1 to ensure a positive relationship with causal gene status. We capped transformed pLI at its 99th percentile (34.3) due to a long tail. We also included binary indicator variables for gene-level covariate missingness, pLI < 0.1, and pLI < 0.9.

### Model training, testing, and performance

To maximize the applicability of model predictions to new traits, we trained models using a nested leave-one-trait-out (LOTO) cross-validation framework. In the outer fold, we held one trait out as a test set. In the remaining 18 traits, we trained LASSO and XGBoost models using an inner fold of LOTO cross-validation to select hyperparameters. We used these trained models to predict causal gene probability in the held-out test set. We then used these predictions to compute AUPRC using the pr.curve function and the auc.integral method from the PPROC R package^34^. We computed AUPRC 95% CIs using the logit method^35^.

### LASSO

We trained LASSO models using the cv.glmnet function from the glmnet R package^36^, selecting the lambda value with the minimum mean cross-validated error. Where specified, we included gene-level covariates when training models, but set covariates values to their mean in the held-out test sets.

### XGBoost

We trained XGBoost models using the xgboost and mlr R packages. We used a binary logistic objective function and 100 hyperparameter sets. For each set, we randomly sampled hyperparameters from uniform distributions (see Table S2 for hyperparameters and their ranges). We did not include gene-level covariates when training or testing XGBoost models.

### Recalibration

We generated calibration plots using the cal_plot_logistic function from the “probably” R package. We locally-recalibrated predictions, once again using a nested LOTO framework. In each outer fold we trained models and used them to generate initial predictions in both the training set and the test set. Next, we trained a second LASSO model to predict causal gene status using the initial training set predictions (on the logit scale), as well as the relative predictions within each locus (focal - best). We applied this model to the initial test set predictions to get recalibrated predictions.

### Recovering known characteristics of GWAS genes

We defined putatively causal CALDERA genes as the set of 149 unique genes with predicted causal probability > 50% for any trait. We defined putatively non-causal CALDERA genes as the remaining 2,043 unique genes in GWAS loci for these traits. Using linear or logistic regression, we tested for an association between putative causal gene status and: 1) pLI > 90%, 2) whether a gene is a transcription factor, and 3) the number of unique TSSs across gene isoforms. We extracted these gene-level features from a study by Mostafavi and colleagues^22^.

### Benchmarking

To compare CALDERA and L2G performance, we used the Open Targets and ExWAS benchmarking datasets from the FLAMES study^6^. We constructed CALDERA features using pre-computed values from the FLAMES study for PoPS (‘PoPS_Score’), MAGMA (‘MAGMA-Z’), distance (‘distance’, also used to compute the number of genes within 300kb of each GWAS signal), and coding PIP (‘VEP_sum’). Note that these coding PIP values are systematically smaller than the ones in the CALDERA truth set because PIP was multiplied by a shrinkage factor based on VEP effect (HIGH = 1, MODERATE = 0.6, LOW = 0.4, MODIFIER = 0.1). AUPRC values for CALDERA and L2G were calculated on the subset of genes with precomputed L2G scores. Seven traits in the ExWAS dataset were identical or highly correlated with traits used to train CALDERA. We therefore used a version of CALDERA excluding these traits (calcium, estimated bone mineral density, hemoglobin, hemoglobin A1c, adult height, low density lipoprotein cholesterol, total bilirubin).

## Supplementary Material

Supplement 1

Supplement 2

## Data Availability

All credible set and variant-to-gene mapping data for UK Biobank traits are available at https://www.finucanelab.org/data. All other data and code required to reproduce these analyses are available on GitHub at https://github.com/kheilbron/caldera. Gencode release 44: https://www.gencodegenes.org/human/release_44.html The Mostafavi *et al*. 2023^22^ Zenodo repository: https://zenodo.org/records/6618073

## Footnotes

Code availability statement CALDERA is available as a set of open-source R scripts at https://github.com/kheilbron/caldera. Additional software and code: MAGMA: https://cncr.nl/research/magma/ PoPS: https://github.com/FinucaneLab/pops
